# Clinical and Radiological Characteristics of Patients with *Lophomonas blattarum* Detected in Bronchial Lavage: A Single-Center Experience

**DOI:** 10.1007/s10096-026-05539-9

**Published:** 2026-05-18

**Authors:** Demet Polat Yulug, Hakan Dedeoglu, Fatma Sevval Unal, Nurbanu Yasar, Damla Hazal Sucu, Nuran Delialioglu, Sibel Nayci, Eylem Sercan Ozgur

**Affiliations:** 1 Department of Chest Diseases, Faculty of Medicine, Mersin University, Mersin, Turkey; 2 Department of Radiology, Faculty of Medicine, Mersin University, Mersin, Turkey; 3 Department of Medical Microbiology, Faculty of Medicine, Mersin University, Mersin, Turkey; 4 Department of Biostatistics, Faculty of Medicine, Mersin University, Mersin, Turkey

**Keywords:** Lophomonas blattarum, Bronchial lavage, Bronchoscopy, Respiratory specimens, Protozoan detection, Computed tomography

## Abstract

**Introduction:**

*Lophomonas blattarum (L. blattarum)* is an emerging protozoan increasingly reported in respiratory specimens. This study aimed to characterize the clinical and radiological features of adult patients with *L. blattarum* detected in bronchial lavage samples.

**Methods:**

This retrospective observational study included adult patients diagnosed at a tertiary care center between January 1, 2024 and January 1, 2026. Demographic data, comorbidities, clinical presentation, laboratory findings, radiological features, treatment approaches, and outcomes were analyzed descriptively.

**Results:**

Fifty-one patients were included (mean age 59.0 ± 14.8 years; 51.0% male). Symptoms were predominantly chronic, with 73.3% reporting duration > 8 weeks. The most common symptoms were cough (73.3%), sputum (46.7%), and dyspnea (45.7%); fever was uncommon (6.5%). Frequent comorbidities included diabetes mellitus (31.9%), hypertension (29.8%), cardiovascular disease (23.4%), prior tuberculosis (25.0%), malignancy (26.0%), and immunosuppression (21.3%). More than half were never-smokers (52.2%). The mean leukocyte count was 9.02 ± 3.39 × 10⁹/L, and the median C-reactive protein level was 12.70 mg/L (IQR: 3.38–42.88). Chest CT findings were heterogeneous. Nodules were most frequent (38.3%), followed by ground-glass opacities (29.8%) and small airway signs (27.7%). Consolidation was observed in 16.3% and mediastinal or hilar lymphadenopathy was present in 48.9%. Metronidazole was administered to 54.9% of patients; radiological improvement was observed in 21.7%.

**Conclusion:**

Microscopy-based detection of *L. blattarum*-like organisms in bronchial lavage was mainly observed in patients with chronic respiratory symptoms, heterogeneous and non-specific CT findings, and substantial comorbidity burden. Further prospective controlled studies with molecular confirmation are needed to clarify its clinical significance.

## Introduction

Global climate change, rising temperatures, altered precipitation patterns, and urbanization-driven ecosystem transformations are recognized determinants influencing the epidemiology of infectious diseases. Climate-related environmental changes may affect parasite life cycles and transmission dynamics, thereby contributing to shifts in the geographic distribution of infections [1, 2]. Furthermore, globalization and increasing global mobility—particularly air travel, migration, and armed conflicts—facilitate the introduction of endemic infections into new regions, reshaping the epidemiological landscape of parasitic diseases [1–3].

*Lophomonas blattarum (L. blattarum)* is a multiflagellated parabasalid protozoon originally identified in the gastrointestinal tract of cockroaches [4]. Over the past decade, reports describing its detection in bronchoalveolar lavage (BAL) and other lower respiratory tract specimens have increased substantially. The progressive rise in PubMed-indexed publications supports this observation. A global systematic review including 307 cases reported between 1993 and 2020 demonstrated a marked increase in published cases in recent years, with the majority originating from Asia, particularly Iran [5]. However, cases have also been increasingly reported from Europe, the Americas, and Africa, suggesting a broader geographic distribution. Despite these reports, the true prevalence of *L. blattarum* in respiratory disease remains uncertain. This uncertainty largely stems from reliance on light microscopy for diagnosis and the limited availability of standardized molecular confirmation methods [4, 6–8].

Clinically, detection of *L. blattarum* has been associated with non-specific respiratory symptoms, including chronic cough, sputum production, dyspnea, fever, and recurrent pulmonary infiltrates [4, 9, 10]. In critically ill populations, clinical presentations appear heterogeneous and are frequently accompanied by significant comorbidities [9].

Although the number of publications has increased, the current literature predominantly consists of case reports and small case series [5]. Comprehensive, center-based descriptive studies systematically evaluating demographic characteristics, comorbidity burden, clinical presentation, and radiological patterns in adult patients remain limited.

In the present study, we aimed to characterize the demographic features, clinical findings, comorbidities, and radiological patterns of adult patients with *L. blattarum* detected in bronchial lavage samples at a tertiary care center. Additionally, treatment approaches and short-term clinical outcomes were assessed.

### Materials and Methods

The study protocol was approved by the Ethics Committee of Mersin University Faculty of Medicine. The study was conducted in accordance with the principles of the Declaration of Helsinki. Due to the retrospective design and the use of anonymized medical records, the requirement for written informed consent was waived by the Ethics Committee.

### Study Design and Population

This retrospective, single-center study was conducted at a tertiary care hospital. All adult patients who underwent fiberoptic bronchoscopy (FOB) with bronchial lavage between January 1, 2024 and January 1, 2026 were screened.

During the study period, 51 patients in whom *L. blattarum* was detected in bronchial lavage samples were included in the study.

### Inclusion and Exclusion Criteria

Patients aged ≥ 18 years with documented detection of *L. blattarum* in bronchial lavage samples were included. Patients with incomplete clinical or radiological data were excluded from the analysis. If repeated bronchoscopic procedures were performed in the same patient, only the first episode was included.

### Data Collection

Demographic characteristics, smoking status, comorbidities, presenting symptoms, laboratory findings, and indications for bronchoscopy were obtained from electronic medical records.

Chest computed tomography (CT) scans performed at the time of diagnosis were reviewed through the institutional picture archiving and communication system (PACS). CT images were reviewed by an experienced thoracic radiologist . No formal interobserver agreement analysis was performed.

### Microbiological Evaluation

Bronchial lavage samples were examined by light microscopy in the microbiology laboratory. Microscopic identification was based on morphological features considered compatible with *Lophomonas*-like organisms, including oval or pear-shaped forms, multiple irregularly arranged flagella, granular cytoplasm, and characteristic motility when observed. The preparations were evaluated by an experienced microbiologist. Polymerase chain reaction, electron microscopy, and independent blinded confirmation were not routinely available and were not performed.

### Statistical Analysis

Statistical analyses were performed using SPSS version 26.0 (IBM Corp., Armonk, NY, USA). Continuous variables were expressed as mean ± standard deviation or median with interquartile range, as appropriate. Categorical variables were presented as frequencies and percentages. Percentages were calculated according to the number of patients with available data for each variable.

Radiological findings were compared between subgroups according to immunosuppression status and history of previous tuberculosis. Categorical variables in these subgroup comparisons were analyzed using Pearson’s chi-square test or Fisher’s exact test, as appropriate, depending on expected cell counts. A two-sided p value of < 0.05 was considered statistically significant.

A network graph was generated to visualize multidimensional relationships among selected clinical, laboratory, and radiological variables. Variables included in the network were selected based on clinical relevance and data availability. In the network structure, nodes represented individual variables, and edges represented observed co-occurrence or association patterns between variables. The graph was used as an exploratory visualization tool for pattern recognition and hypothesis generation, rather than for confirmatory inferential statistical testing.

## Results

### Demographic and Clinical Characteristics

A total of 51 patients with *L. blattarum* detected in bronchial lavage samples were included (Table 1). The mean age was 59.0 ± 14.8 years, and 51.0% were male.

**Table 1:** Demographic and clinical characteristics of patients with *Lophomonas blattarum* detected in bronchial lavage (*n* = 51)

Variable	
Age (years), mean *±* SD	59.04 ± 14.81
Male sex, n (%)	26 (51.0%)
Never smoker, n (%)	24 (52.2%)
Comorbidities, n (%)	
Diabetes mellitus	15 (31.9%)
Hypertension	14 (29.8%)
Cardiovascular disease	11 (23.4%)
Previous tuberculosis	12 (25.0%)
Malignancy	13 (26.0%)
Immunosuppression	10 (21.3%)
COPD	7 (14.9%)
Symptoms, n (%)	
Cough	33 (73.3%)
Sputum	21 (46.7%)
Dyspnea	21 (45.7%)
Hemoptysis	13 (28.9%)
Chest pain	10 (22.2%)
Fever	3 (6.5%)
Weight loss	11 (23.9%)
Symptom duration, n (%)	
< 1 week	4 (8.9%)
2–8 weeks	8 (17.8%)
> 8 weeks	33 (73.3%)

Clinical presentation was predominantly chronic and insidious. Notably, 73.3% of patients reported symptoms persisting for more than 8 weeks prior to diagnosis. Cough was the most common symptom (73.3%), followed by sputum production (46.7%) and dyspnea (45.7%). Hemoptysis (28.9%) and weight loss (23.9%) were also relatively frequent. In contrast, fever was uncommon, observed in only 6.5% of cases, highlighting the largely non-acute and atypical clinical profile.

Patients demonstrated a considerable comorbidity burden (Table 1). Diabetes mellitus (31.9%), hypertension (29.8%), cardiovascular disease (23.4%), and prior tuberculosis (25.0%) were common. A history of malignancy was present in 26.0%, and 21.3% had underlying immunosuppression. More than half of the patients were never-smokers (52.2%), whereas 23.9% were active smokers.

At diagnosis, the mean leukocyte count was 9.02 ± 3.39 × 10⁹/L and the mean lymphocyte count was 2.04 ± 0.99 × 10⁹/L. The median eosinophil count was 0.22 (IQR 0.10–0.39) ×10⁹/L. The median C-reactive protein level was 12.70 mg/L (IQR 3.38–42.88), and the median erythrocyte sedimentation rate was 34.0 mm/h (IQR 17.0–61.0) (Table 2).

**Table 2:** Laboratory, radiological characteristics and outcomes of patients with *Lophomonas blattarum* detected in bronchial lavage (*n* = 51)

Laboratory Findings	
White blood cell count (×10⁹/L), mean ± SD	9.02 ± 3.39
Eosinophil count (×10⁹/L), median (IQR)	0.22 (0.10–0.39)
Lymphocyte count (×10⁹/L), mean ± SD	2.04 ± 0.99
C-reactive protein (mg/L), median (IQR)	12.70 (3.38–42.88)
Erythrocyte sedimentation rate (mm/h), median (IQR)	34.0 (17.0–61.0)
Radiological findings (Chest CT), n (%)	
Nodules	19 (38.3%)
Ground-glass opacities	15 (29.8%)
Tree-in-bud pattern	14 (27.7%)
Bronchial wall thickening	14 (27.7%)
Consolidation	8 (16.3%)
Mediastinal or hilar lymphadenopathy	24 (48.9%)
Bronchiectasis	4 (7.8%)

Despite the often subacute-to-chronic presentation, clinical severity was substantial. Hospitalization was required in 43.1% of patients, and 5.9% required intensive care unit admission. The all-cause mortality rate reached 11.8%, underscoring the potentially serious clinical course in this population.

## Radiological Findings

Chest CT findings were heterogeneous and non-specific (Table 2). Pulmonary nodules were the most frequent abnormality (38.3%), followed by ground-glass opacities (29.8%). Signs of small airway involvement, including tree-in-bud patterns and bronchial wall thickening, were observed in 27.7% of cases. Consolidation was present in 16.3% of patients. Lower lobe involvement was common (left lower lobe: 46.8%; right lower lobe: 44.7%), as was right upper lobe involvement (40.4%). Mediastinal or hilar lymphadenopathy was identified in 48.9% of patients, most frequently in the mediastinum.

Radiological findings were further evaluated according to immunosuppression status and history of previous tuberculosis. In the subgroup analysis by immunosuppression status, consolidation was significantly more frequent in immunosuppressed patients than in non-immunosuppressed patients, whereas other CT findings did not differ significantly between the groups (Table 3). When radiological findings were compared according to previous tuberculosis history, no statistically significant differences were observed in nodules, ground-glass opacities, tree-in-bud pattern, bronchial wall thickening, consolidation, mediastinal or hilar lymphadenopathy, or bronchiectasis (Table 4).

**Table 3:** Radiological findings stratified by immunosuppression

Radiological findings (Chest CT), *n *(%)	Non-immunosuppressed group *n*:35	Immunosuppressed group *n*:10	*P* value
Nodules	14 (40.0)	4 (40.0)	1.000
Ground-glass opacities	10 (28.6)	4 (40.0)	0.491
Tree-in-bud pattern	8 (22.9)	3 (30.0)	0.643
Bronchial wall thickening	11 (31.4)	3 (30.0)	0.931
Consolidation	1 (2.9)	6 (60.0)	**< 0.001**
Mediastinal or hilar lymphadenopathy	17 (48.6)	5 (50.0)	0.936
Bronchiectasis	3 (8.5)	1 (10.0)	0.252

Percentages were calculated according to the number of patients with available data in each subgroup. Although immunosuppression status was available in 47 patients in the overall cohort, 2 patients had incomplete radiological data and were therefore excluded from the radiological subgroup analysis

**Table 4:** Radiological findings stratified by history of tuberculosis

Radiological Findings (Chest CT), *n *(%)	No previous tuberculosis, *n*:37	Previous tuberculosis*, *n*: 10	*P* value
Nodules	17 (45.9)	2 (20.0)	0.138
Ground-glass opacities	14 (37.8)	1 (10.0)	0.094
Tree-in-bud pattern	10 (27.0)	3 (30.0)	0.852
Bronchial wall thickening	11 (29.7)	3 (30.0)	0.884
Consolidation	6 (16.2)	2 (20.0)	0.778
Mediastinal or hilar lymphadenopathy	19 (51.4)	3 (30.0)	0.940
Bronchiectasis	3 (8.1)	1 (10.0)	0.218

* Percentages were calculated according to the number of patients with available radiological data in each subgroup. Although previous tuberculosis history was documented in 12 patients in the overall cohort, 2 of these patients had incomplete radiological data and were therefore excluded from the radiological subgroup analysis

**Fig. 1:**
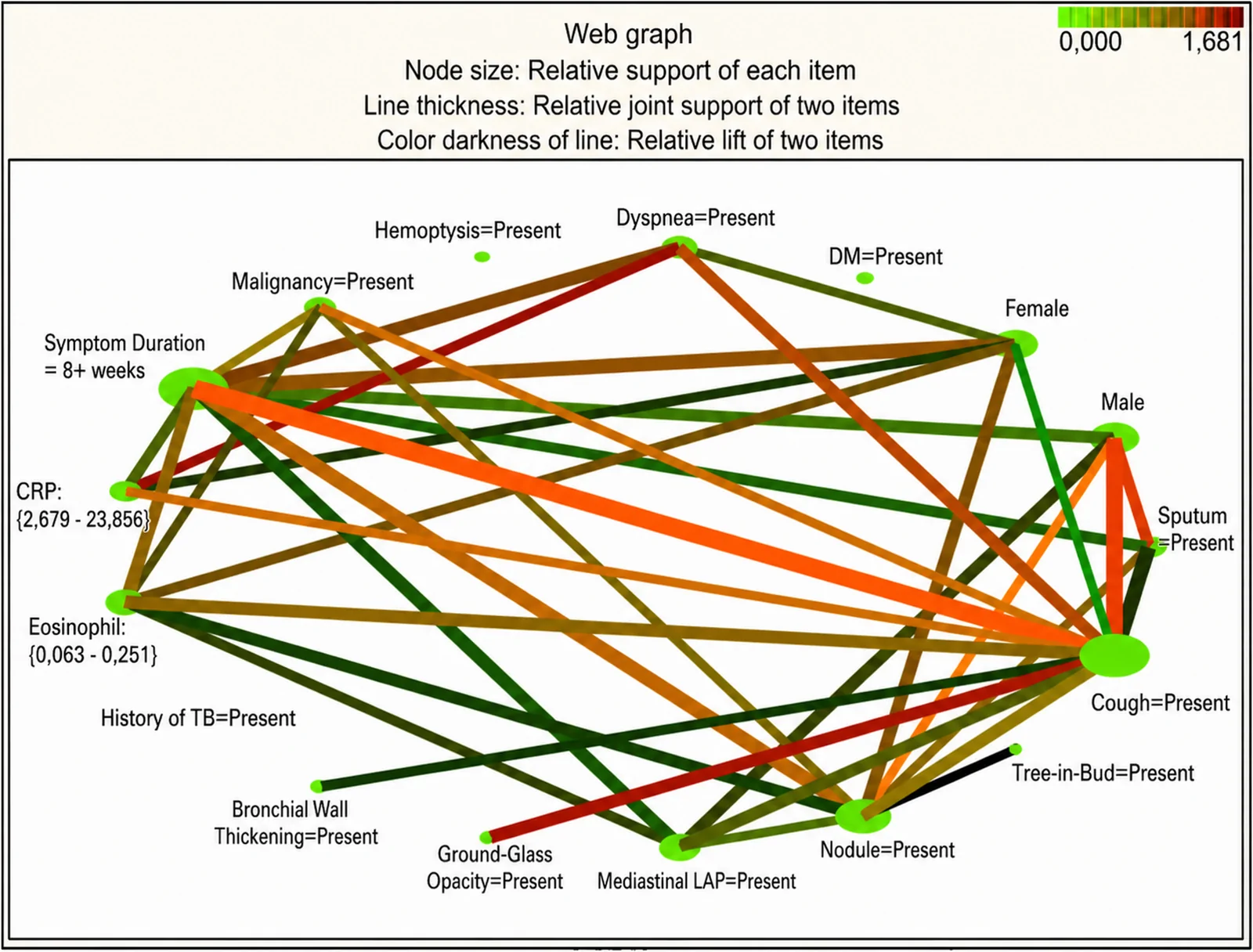
This network graph visualizes the multidimensional relationships among clinical, laboratory, and radiological variables related to the respiratory system. The findings show that key symptoms such as cough and sputum production occupy a central position, while chronic symptom duration and inflammatory markers demonstrate strong connections with this core structure. Radiological findings, in contrast, are more heterogeneously distributed around this clinical framework. Such network representations may serve as valuable analytical tools for pattern recognition, hypothesis generation, and the identification of potential clinical-radiological clusters in complex clinical datasets

A network graph was generated to visualize multidimensional relationships among selected clinical, laboratory, and radiological variables. The graph demonstrated that cough and sputum production occupied a central position, whereas radiological findings were more heterogeneously distributed around this clinical framework (Fig. 1).

### Treatment and Outcomes

Metronidazole-based antiparasitic therapy was administered to 54.9% of patients. Overall clinical and/or radiological response was documented in 27.5% of cases. However, radiological improvement alone was observed in only 21.7% of patients, indicating a limited objective imaging response despite treatment.

## Discussion

In this study, the clinical characteristics, radiological findings, and outcomes of adult patients with microscopy-based detection of *Lophomonas*-like organisms in bronchial lavage samples were systematically evaluated. A substantial proportion of patients presented with chronic respiratory symptoms, elevated inflammatory laboratory markers, heterogeneous CT abnormalities, and relevant underlying comorbidities. Given the absence of molecular confirmation, lack of a comparator group, and non-protocolized treatment decisions these findings should be interpreted as descriptive and hypothesis-generating rather than as evidence of confirmed infection, disease specificity, or treatment efficacy. To the best of our knowledge, this study represents one of the few real-world adult cohorts describing the clinical and radiological profile of patients with microscopy-based detection of *Lophomonas*-like organisms in bronchial lavage samples.

One of the most striking features of the study cohort was the prolonged duration of symptoms in the majority of patients and the low frequency of fever. This pattern suggests a chronic inflammatory or structural lung disease background rather than an acute infectious process. In the literature, many reported cases of *L. blattarum* detection have similarly presented predominantly with chronic cough and sputum production, while specific systemic signs of infection were often absent [4, 9]. Our findings are consistent with these observations.

The high burden of comorbidities is noteworthy. Rates of diabetes mellitus, malignancy, and immunosuppression were considerable. This finding suggests that the microorganism may be more frequently detected in the context of underlying disease. A recent study conducted in a critical care population also emphasized that the clinical features of patients with detected *L. blattarum* are heterogeneous and that careful evaluation is required to determine whether the organism represents a true pathogen or secondary colonization [9]. In our cohort, the relatively high mortality and hospitalization rates do not necessarily indicate that the detected microorganism directly determined clinical severity; rather, they suggest that the underlying disease burden likely played a substantial role.

Radiological assessment did not reveal a specific phenotype. The most common findings were nodules and ground-glass opacities, with an apparent predominance of airway-centered patterns. However, alveolar inflammatory and mixed patterns were also observed at meaningful rates, indicating the absence of a single characteristic CT feature. Previously reported series have likewise described heterogeneous findings, including nodules, ground-glass opacities, and consolidations, without identifying a distinctive radiological pattern [11]. This heterogeneity strengthens the possibility that *L. blattarum* may be detected on the background of pre-existing pulmonary pathology rather than acting as a primary infectious agent. Nevertheless, radiological regression observed in a subset of patients after treatment should be interpreted cautiously, as imaging changes may have been influenced by the natural course of the underlying disease, concomitant therapies, or non-standardized treatment decisions.

In a retrospective pediatric series including fifty-three children, Ding and Shen reported predominantly chronic respiratory symptoms, frequent radiological abnormalities, and complete clinical recovery following metronidazole therapy [12]. In contrast to pediatric reports describing favorable outcomes after metronidazole therapy, only a minority of patients in our cohort demonstrated objective radiological improvement. This difference may reflect the adult population, high comorbidity burden, underlying structural lung disease, and non-standardized treatment decisions. Therefore, the observed improvement after metronidazole should be interpreted cautiously and should not be considered evidence of treatment efficacy or organism pathogenicity. A recent study conducted in a critical care population also reported that detection of *L. blattarum* in patients with severe underlying disease may be associated with adverse clinical outcomes [9]. However, the high mortality and hospitalization rates observed in our cohort cannot be directly attributed to the organism and more likely reflect the substantial burden of underlying comorbidities and baseline disease severity in this selected bronchoscopy population.

From a diagnostic perspective, the main limitation of this study is the reliance on light microscopic morphology without molecular confirmation. The diagnosis of *L. blattarum* remains controversial because detached or degenerated ciliated bronchial epithelial cells may mimic flagellated protozoa, and differentiation requires expertise and may be subject to observer variability. Although molecular confirmation would have strengthened diagnostic validity, currently available PCR-based approaches are not fully standardized, and false-positive or non-specific amplification has been reported. In particular, Lee et al. showed that the current diagnostic scheme and PCR testing may lead to false-positive results in bronchoalveolar lavage samples containing *Lophomonas*-like structures [13]. Similarly, in a previously reported case from our center, PCR amplification yielded non-specific products with high similarity to the human genome [14]. Therefore, our findings should be interpreted as microscopy-based detections of *Lophomonas*-like organisms rather than microbiologically confirmed infections. Another important limitation is the absence of a comparator group. Since BAL-negative patients with similar clinical profiles were not included, it is not possible to determine whether the observed clinical and radiological findings are specific to *Lophomonas* detection, incidental, or mainly related to underlying pulmonary disease. The retrospective, single-center design, lack of standardized radiological scoring, non-protocolized treatment decisions, incomplete follow-up imaging, and absence of independent blinded confirmation by multiple observers further limit causal interpretation. Nevertheless, this study presents a relatively large adult cohort with systematically collected clinical, laboratory, radiological, and outcome data.

Microscopy-based detection of *L. blattarum*-like organisms in bronchial lavage was observed mainly in patients with chronic respiratory symptoms, heterogeneous and non-specific CT findings, and a substantial comorbidity burden. In the absence of molecular confirmation and a comparator group, these findings should not be interpreted as evidence of confirmed *L. blattarum* infection, disease specificity, or treatment efficacy. Further prospective, controlled studies incorporating standardized diagnostic methods, including molecular or ultrastructural confirmation, are needed to clarify the clinical significance of this finding.

## Data Availability

No datasets were generated or analysed during the current study.
